# Lem3, a phospholipid flippase subunit, influences *Candida albicans* pathogenicity through maintenance of plasma membrane asymmetry

**DOI:** 10.1128/spectrum.02965-25

**Published:** 2026-03-31

**Authors:** Pranjali Agrawal, Pritam Kumar, Basharat Ali, Sunna Nabeela, Mélanie A. C. Ikeh, Joachim Morschhäuser, David R. Andes, Clarissa J. Nobile, Rajendra Prasad, Priya Uppuluri, Sneh Lata Panwar

**Affiliations:** 1 Yeast Molecular Genetics Laboratory, School of Life Sciences, Jawaharlal Nehru University 124711 , New Delhi, India; 2 Amity Institute of Biotechnology, Amity University Gurugram 307019, Gurugram, Haryana, India; 3 Division of Infectious Diseases, The Lundquist Institute for Biomedical Innovation at Harbor-University of California Los Angeles Medical Center 117316, Torrance, California, USA; 4 Department of Molecular and Cell Biology, University of California 33244, Merced, California, USA; 5 Institute of Molecular Infection Biology, University of Würzburg, Würzburg, Germany; 6 Department of Medicine, Section of Infectious Diseases, University of Wisconsin 200889, Madison, Wisconsin, USA; 7 Health Sciences Research Institute, University of California 33244, Merced, California, USA; CNRS-Inserm-Université Côte d'Azur, Nice, France

**Keywords:** P4-type ATPases, flippase, Lem3, *Candida albicans*, virulence, biofilms, oropharyngeal candidiasis

## Abstract

Flippases, composed of catalytic and regulatory subunits, preserve cellular integrity by maintaining the asymmetric distribution of phospholipids across the plasma membrane. Here, we provide the first functional characterization of Lem3, the regulatory subunit of the flippase complex in *Candida albicans*, and reveal its critical role in membrane homeostasis and pathogenesis. We demonstrate that Lem3 localizes to both the endoplasmic reticulum and plasma membrane, consistent with its role in phospholipid translocation. Phenotypic analysis of a *lem3*Δ/Δ mutant showed decreased susceptibility to the alkylphosphocholine analog, miltefosine. Concurrently, the mutant was defective in the inward translocation (flip) of phosphatidylcholine (PC) and phosphatidylethanolamine (PE) across the plasma membrane, accompanied by altered levels of ergosterol and sphingolipids, indicating the requirement of Lem3 for maintaining membrane lipid composition. Notably, *LEM3* deletion suppressed the enhanced phospholipid internalization seen in an *rta3*Δ/Δ mutant, suggesting that Rta3 negatively regulates Lem3 activity. The *lem3*Δ/Δ strain exhibited increased susceptibility to azole antifungals, likely due to increased membrane permeability and impaired function of the major drug efflux pump Cdr1. Strikingly, the absence of *LEM3* reversed drug resistance in an azole-resistant clinical isolate, reinforcing its therapeutic relevance. Finally, Lem3 was important for biofilm formation and *in vivo* virulence, establishing it as a key determinant of *C. albicans* pathogenicity. Together, these findings position Lem3 and the flippase machinery as promising targets for antifungal therapy.

**IMPORTANCE:**

Plasma membrane lipid composition, maintained by lipid translocators, is a key determinant of virulence in pathogenic fungi. Here, we demonstrate that Lem3, the regulatory subunit of the flippase complex, by regulating the asymmetric distribution of phospholipids, impacts the expression of virulence traits and modulates drug resistance in *Candida albicans*. These findings highlight how diverse cellular processes are interconnected through flippase-mediated lipid homeostasis. With the alarming rise in fungal infections and antifungal resistance, targeting lipid translocators such as Lem3 offers a promising avenue for the development of novel therapeutic targets.

## INTRODUCTION

Phospholipids in eukaryotic cells are asymmetrically distributed across the plasma membrane bilayer. The cytoplasmic leaflet of the plasma membrane is enriched with the aminophospholipids, phosphatidylethanolamine (PE), and phosphatidylserine (PS), whereas phosphatidylcholine (PC) and sphingolipids are restricted to the exoplasmic leaflet (1, 2). Given the amphipathic nature of phospholipids, their transbilayer movement is mediated by enzymes, namely, flippases, floppases, and scramblases. Flippases and floppases mediate ATP-dependent inward and outward movement of phospholipids, respectively (1, 3). In contrast, scramblases enable energy-independent, bidirectional phospholipid redistribution between the two monolayers (4). Balancing the activities of these enzymes is pivotal for maintaining the asymmetric distribution of lipids at the plasma membrane and intracellular organelle membranes. A large number of ABC transporters function as floppases, whereas the P4-type ATPases, exclusively present in eukaryotes, serve as flippases. P-type ATPases are a family of membrane proteins, divided into five subfamilies (P1–P5), deriving energy from ATP hydrolysis to facilitate the transverse movement of phospholipids (5). The activity of these proteins is regulated by phosphorylation at an aspartate residue, a feature that distinguishes them from other ATP-dependent transporters (6).

In *Saccharomyces cerevisiae,* four of the five P4-ATPases (Drs2 and Dnf1–3) associate with the non-catalytic regulatory β-subunits of the Cdc50/Lem3 family (Cdc50, Lem3, and Crf1), which are essential for ER exit, localization, and flippase function; the fifth P4-ATPase, Neo1, functions independently of these subunits (7–9). These flippase complexes are distinct in their lipid substrate profile and subcellular localization. Drs2/Cdc50 functions at the trans-Golgi network (TGN) and secretory vesicles to flip PS and PE, Dnf2 and Dnf1 associate with Lem3 at the plasma membrane to flip PC, PE, and glucosylceramide (GlcCer) and regulate endocytosis, while Dnf3 localizes to the TGN and reaches the plasma membrane in a cell cycle-dependent manner to direct the flip of PS/PE (9–13). Combined together, these complexes are required for maintaining membrane curvature and membrane trafficking pathways (14).

P4-ATPases are conserved among various pathogenic fungi, including the commensal-pathogen, *Candida albicans*. This fungus resides as a harmless member of the human mucosal and skin microbiota. In immunocompetent individuals, the fungus causes superficial infections, whereas in immunocompromised individuals, it can lead to systemic infections (15). The ability of *C. albicans* to switch from yeast form to hyphal form, a crucial virulence trait, facilitates its survival within the mammalian host. The transport of virulence factors is dependent on vesicle budding during endocytosis and the secretory pathway. These processes require intense membrane remodeling, which is facilitated by the P4-ATPases, thus ensuring the maintenance of lipid asymmetry. Consequently, virulence factors are properly targeted to the exoplasmic side of the plasma membrane for biofilm formation and morphogenesis. Several studies have demonstrated a connection between maintenance of plasma membrane asymmetry and the fungus’ ability to undergo hyphal morphogenesis to establish infection. In *C. albicans*, PS and PI(4)P (phosphatidylinositol 4-phosphate) distribution is altered in *drs2* mutants, whereas *dnf2* mutants are unable to flip glucosylceramide (GlcCer)/PC/PE, which may lead to defects in endocytosis or exocytic vesicle budding, resulting in defective hyphal growth (10, 15–17). Deletion of *DRS2* also results in increased susceptibility to copper ions, Calcofluor white (CFW), fluconazole, and SDS (17). Furthermore, deletion of the associated β-subunit Cdc50 impacts virulence and antifungal resistance in several fungal species, including *C. albicans*, *Nakaseomyces glabratus*, and *Cryptococcus neoformans* (18–22). Similarly, DnfB, the Drs2 homolog in *Aspergillus nidulans*, is involved in PS flipping and hyphal growth (23). Among Dnf1-3, only Dnf2 is required for flipping GlcCer, PC, and PE and impacts virulence in *C. albicans* (16). Lem3, the regulatory subunit of Dnf2, and Dnf1 remain uncharacterized in *C. albicans*. Given this gap, we investigated the contribution of Lem3 to membrane lipid homeostasis in *C. albicans*. We show that Lem3 localizes to both the ER and plasma membrane, consistent with a role in phospholipid flipping. A *lem3*Δ/Δ mutant displayed defective inward translocation of PC and PE, altered sterol and sphingolipid content, and decreased susceptibility to the alkylphosphocholine analog miltefosine, indicating the involvement of Lem3 in maintaining membrane lipid composition by virtue of its ability to regulate the transbilayer movement of PC/PE. Consequently, the disruption in membrane lipid organization resulted in increased membrane permeability and impaired efflux activity of the ABC transporter Cdr1, leading to enhanced susceptibility to azole antifungals. Importantly, *LEM3* deletion reversed drug resistance in a clinical azole-resistant isolate, underscoring its potential as a therapeutic target. To further probe Lem3’s flippase activity, we used an *rta3*Δ/Δ mutant, which exhibits enhanced phospholipid internalization (24). Deletion of *LEM3* in this background (i.e., *rta3*Δ/Δ*lem3*Δ/Δ) abrogated the *rta3*Δ/Δ-associated phenotypes, suggesting that Rta3 acts as a negative regulator of Lem3 function. Finally, Lem3 was essential for the expression of key virulence traits because the *lem3*Δ/Δ mutant was defective in biofilm formation and failed to establish an infection *in vivo*. Taken together, this is the first study to characterize Lem3 in *C. albicans*. Our findings reveal that Lem3 is a central regulator of membrane asymmetry, drug susceptibility, and virulence and highlight the therapeutic potential of targeting the flippase complex for antifungal therapy.

## RESULTS

### Lem3 is an ER-localized protein regulating the transbilayer lipid arrangement in the plasma membrane

**Fig 1:**
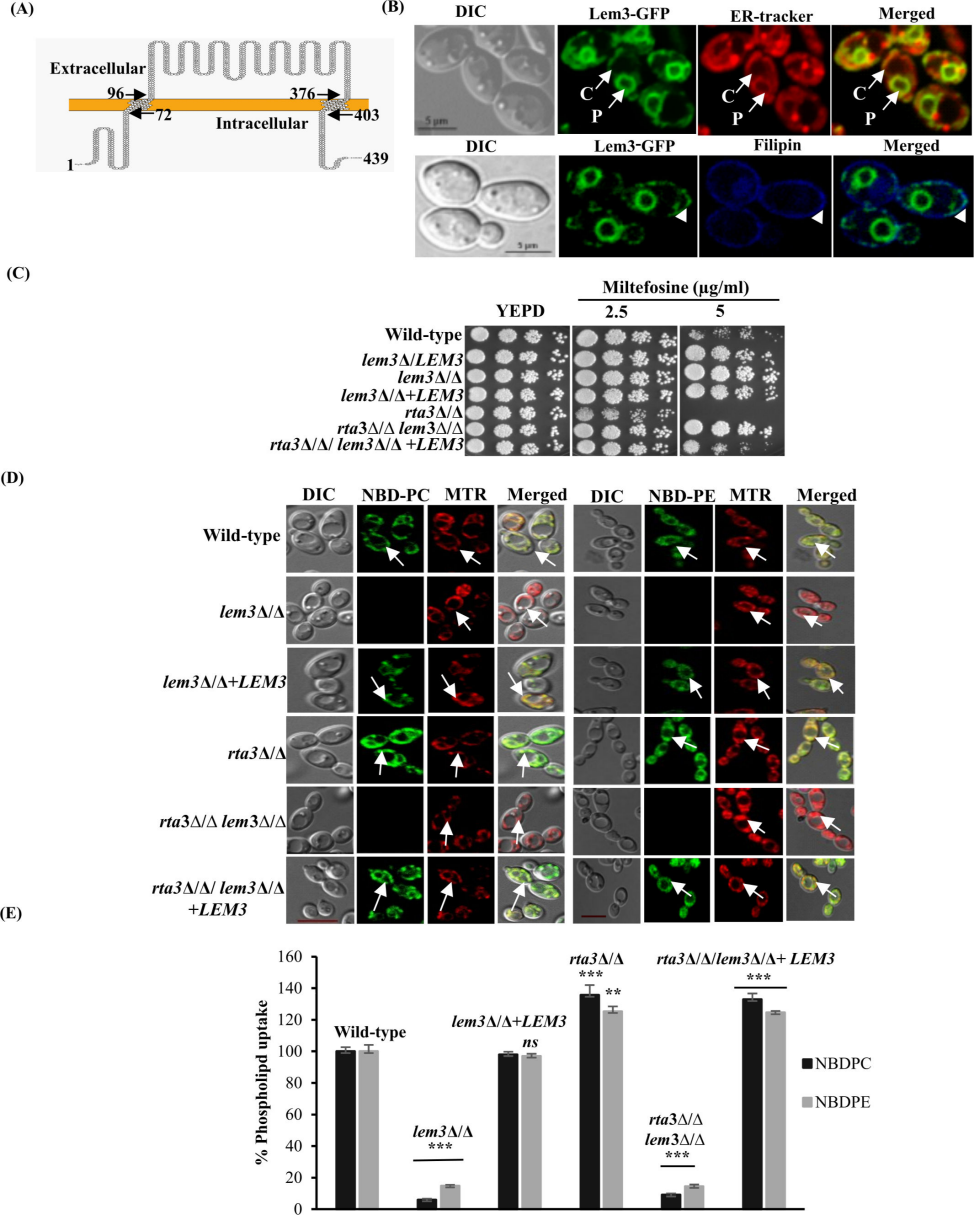
Lem3 regulates the transbilayer lipid arrangement at the plasma membrane. (**A**) A schematic representation of Lem3 containing transmembrane domains spanning amino acids 72–96 and 376–403 at the N- and C-termini, respectively. (**B**) Cells expressing Lem3-GFP were grown to early log phase, co-stained with ER Tracker Red and filipin for confocal microscopy. Arrows (upper panel) and arrowheads (lower panel) indicate the cortical (C) and perinuclear (P) ER and plasma membrane localization of Lem3, respectively. Gamma correction (0.7) was applied to both panels to better represent the dimly labeled cells. (**C**) Fivefold serial dilutions of cell suspensions were spotted on YEPD plates with specified drug concentrations and incubated at 30°C for 48 h. (**D**) Strains were grown to an OD_600_ of 1, labeled with 5 µM NBD-lipids, and co-stained with 200 nM MitoTracker Red (MTR) for 45 min at 30°C. Cells were incubated in fresh SDC medium (30 min, 30°C), washed with ice-cold SC-azide. Approximately 200 cells were visualized using confocal microscopy. Arrows indicate mitochondria localized to NBD-lipids. Scale bars are 5 μm. (**E**) Uptake of NBD-lipids was quantified by fluorescence intensity as measured by FACS and normalized to the wild type, which was defined as 100%. All other values are expressed as a percentage of wild-type uptake (*X*/WT × 100; *X* = value of respective strain). Data represent the mean ± SD of three independent experiments, validated by Student’s *t*-test (ns; not significant; **P < 0.01, ***P < 0.001).

BLAST analysis shows the existence of five P4-ATPases in the *C. albicans* genome (16). These ATPases contain α (Dnf1-3, Drs2, and Neo1) and β (Cdc50, Lem3, and Crf1) subunits, identified on the basis of sequence homology. Sequence analysis predicted that *LEM3* encodes a 439-amino-acid (67 kDa) protein containing two putative transmembrane domains near the N-terminus (residues 73–95) and C-terminus (residues 377–402) along with a large, exoplasmic domain (Fig. 1A). To determine the cellular localization of Lem3, we tagged the gene sequence with GFP and replaced the endogenous promoter with that of *TDH3* (25, 26). Lem3 was localized in the ER and the plasma membrane, consistent with the appearance of fluorescence in the perinuclear and cortical ER, and as a continuous ring at the periphery of the cell, respectively (Fig. 1B).

In *S. cerevisiae*, Lem3 was initially identified for its role in resistance to the alkylphosphocholine drugs miltefosine and edelfosine (9). Similarly, a *C. albicans lem3*Δ/Δ mutant displayed resistance to miltefosine without fitness defects, suggesting functional conservation of Lem3 between the two fungi (Fig. 1C). The reconstituted strain exhibited partial reversion to a phenotype that is somewhere between the wild type and the null mutant, indicating a gene dosage effect of *LEM3*. Resistance to miltefosine is associated with defects in the internalization of its fluorescent structural analog, NBD-labeled PC, in *S. cerevisiae* and *C. albicans* (24, 27). Therefore, to explore the relationship between Lem3 and phospholipid uptake, we measured the internalization dynamics of NBD-labeled lipids (NBD-PC and NBD-PE) in the *lem3*Δ/Δ mutant. Strains grown to an OD_600_ of 1 were incubated with NBD-PC or NBD-PE for 45 min at 30°C in SDC media and subsequently washed with ice-cold SC-azide (synthetic complete) before being subjected to confocal microscopy and flow cytometry. NBD-lipids were primarily associated with mitochondria as indicated by co-localization with mitotracker red (Fig. 1D). The absence of intracellular fluorescence in the *lem3*Δ/Δ mutant indicated a defect in internalization of PC and PE. The *lem3*Δ/Δ mutant showed a 90% reduction in the number of cells that accumulated NBD-PC/PE as deduced from flow cytometric analysis, compared with the wild-type and the reconstituted strains (Fig. 1E).

To further affirm the role of Lem3 as a lipid translocator, we included an *rta3*Δ/Δ strain in our data set, as the mutant exhibits (i) increased susceptibility to miltefosine and (ii) enhanced intracellular accumulation of NBD-PC and NBD-PE (Fig. 1C through E), albeit to different extents, as demonstrated in an earlier study (24). Rta3 is a 7-transmembrane receptor protein that regulates the asymmetric distribution of phospholipids across the plasma membrane and is crucial for biofilm formation. We posit that, if the enhanced intracellular fluorescence associated with *rta3*Δ/Δ cells was due to Lem3-mediated flip of phospholipids, the deletion of *LEM3* in the *rta3*Δ/Δ mutant should reduce the intracellular accumulation of NBD-PC/PE in the *rta3*Δ/Δ/*lem3*Δ/Δ (double mutant) cells. Consistently, the double mutant exhibited decreased susceptibility to miltefosine, whereas reconstituting the strain with *LEM3* resulted in a phenotype that was intermediate between the *rta3*Δ/Δ and *lem3*Δ/Δ mutant, reaffirming the gene dosage effect of *LEM3* (Fig. 1C). Moreover, the double mutant was devoid of intracellular fluorescence for both NBD-labeled lipids, confirming the role of Lem3 in regulating the inwardly directed transbilayer movement of phospholipids in the wild-type as well as the *rta3*Δ/Δ cells (Fig. 1D and E). The total cellular phospholipid levels remained unaltered in the *lem3*Δ/Δ cells (data not shown), restricting the role of Lem3 to regulating the transbilayer movement of phospholipids across the plasma membrane. Overall, these data support the view that Lem3 serves as the lipid translocator to maintain the asymmetric distribution of phospholipids (PC/PE), and Rta3 may be regulating the flippase function of Lem3 in *C. albicans*.

### *lem3*∆/∆ cells display altered membrane permeability associated with perturbations in membrane lipid composition

**Fig 2:**
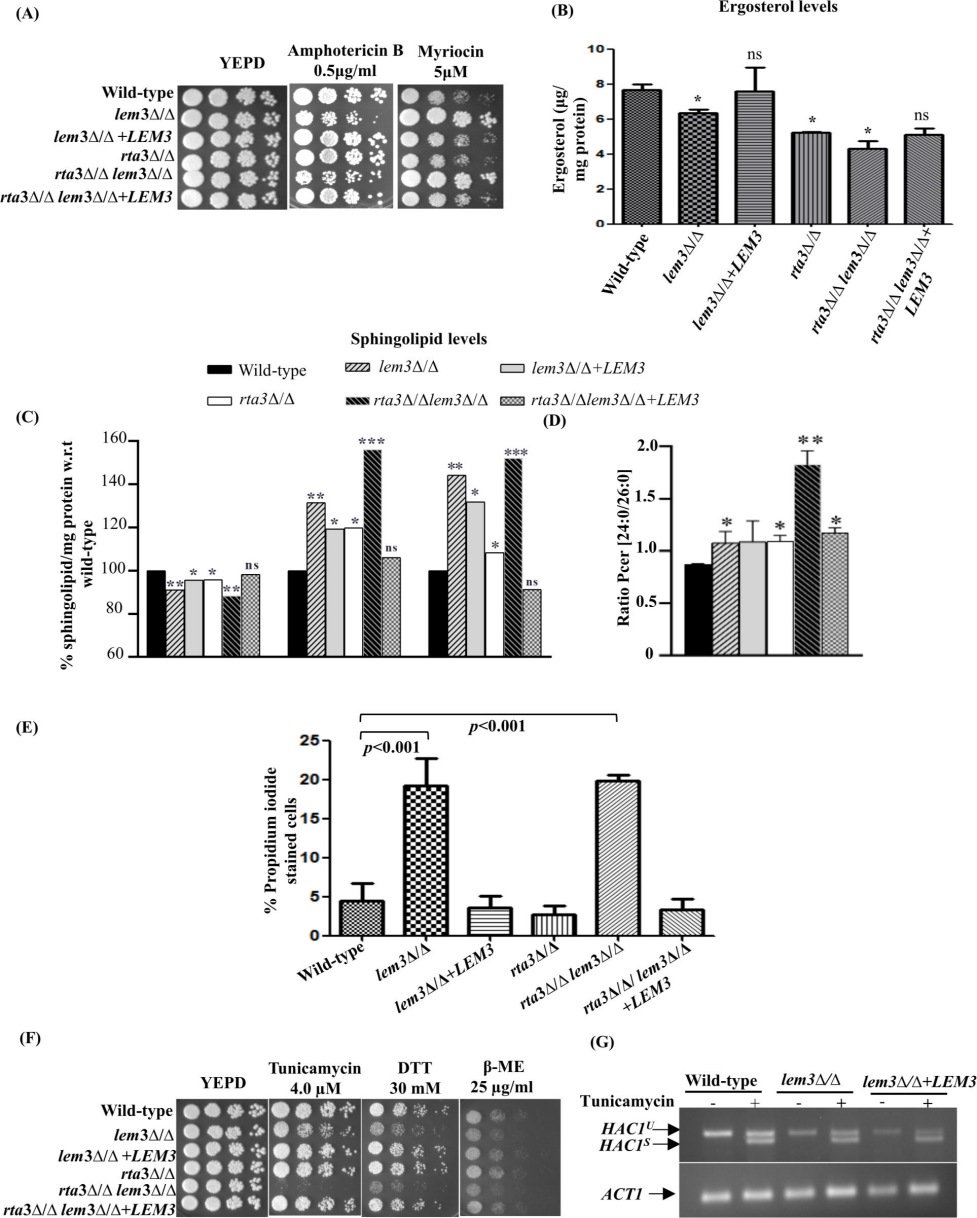
Lem3 impacts membrane lipid composition. (**A**) Fivefold serial dilutions of strains were spotted onto the indicated drug plates and incubated for 48 h at 30°C. (**B**) Ergosterol content of strains, quantified by GC-MS. (**C**) SL quantification of strains was done using the internal standard normalization method. The proportion of each SL class was calculated as % of the total SL mass spectral signal in three biological replicates. Bar graphs represent the % change in each class compared to the wild type. (**D**) Ratio of major SL molecular species PCer 24:0/PCer 26:0; PCer; phytoceramide, α-OH Pcer; hydroxyphytoceramide, α-OH dhCer; hydroxydihydroceramide, α-OH Cer; hydroxyceramide. (**E**) The indicated strains were incubated with propidium iodide (PI; 6 μg/mL, 20 min), washed with PBS, followed by flow cytometry analysis. PI uptake was quantified as the percentage of fluorescence intensity in the respective strains. (**F**) Fivefold serial dilutions of the indicated strains were spotted onto drug-containing plates and incubated for 48 h at 30°C. (**G**) RT-PCR analysis showing *HAC1* mRNA splicing. cDNA was prepared from the indicated strains grown in YEPD media, with and without tunicamycin (4.73 µM, 2 h) treatment. *HAC1^U^*-unspliced mRNA; *HAC1^S^*-spliced mRNA. Data represent means ± SD from three independent assays, validated by Student’s *t*-test in all experiments (*P < 0.05; **P < 0.01; ***P < 0.001; ns; not significant)*.*

The affinity of the phospholipids for sterols depends on the size of their polar head group and the degree of unsaturation in the fatty acyl chains. This interaction is a determinant of the various properties of the plasma membrane, such as its packing, permeability, and rigidity. Additionally, the activity of the phospholipid flippases is linked to sterol metabolism and localization (28). To probe the effect on the activity of the sterol and sphingolipid (SL) biosynthetic pathways in the *lem3*Δ/Δ mutant, we used amphotericin B, which binds ergosterol, and myriocin, an inhibitor of serine palmitoyl transferase in the SL pathway. Spot assays revealed increased susceptibility of the *lem3*Δ/Δ and the double mutant to amphotericin B and decreased susceptibility to myriocin, supporting the idea that the absence of Lem3 affects membrane lipid composition. In contrast, *rta3*∆/∆ cells exhibited wild-type levels of susceptibility to both drugs (Fig. 2A). To further correlate the altered distribution of PC/PE across the plasma membrane with sterol levels, we subjected the various mutants to GC-MS analysis. The *lem3*∆/∆ cells exhibited a 25% (6 μg/mg protein) decrease in ergosterol, compared to the wild-type (8 μg/mg protein) and the reconstituted strains (8 μg/mg protein), a finding consistent with the impact of flippases on sterol homeostasis. Moreover, the *rta3*∆/∆ cells showed a 37% (5 μg/mg protein) decrease in the ergosterol levels with a commensurate reduction of 46% (4.3 μg/mg protein) in the *rta3*Δ/Δ *lem3*Δ/Δ cells. The double mutant reconstituted with *LEM3* exhibited ergosterol levels similar to *rta3*∆/∆ cells (Fig. 2B). Given the coordination between the ergosterol and the SL biosynthesis pathways, we assumed that altered ergosterol levels would impact SL levels in the mutants. Therefore, we sought to determine the levels of SL biosynthesis pathway intermediates by using targeted electrospray ionization-based liquid chromatography tandem mass spectrometry (ESI-LCMS/MS) (29). The analysis revealed that phytoceramide levels (PCer) decreased in the *lem3*Δ/Δ (9%), *rta3*Δ/Δ (5%), and the double mutant (12%), whereas the mutants exhibited significant increases ranging between 8% and 60% in α-hydroxy ceramides and α-hydroxy phytoceramides, compared to the reference strain (Fig. 2C). Next, we analyzed the variation in the molecular species of the various SL intermediates by monitoring changes in fatty acid chain length, degree of saturation, and types of head group, etc. Strikingly, the ratio of two major molecular species, PCer 24:0 and PCer 26:0, changed significantly between the strains. For instance, the ratio of PCer24:0/PCer26:0 was ~0.8 in the wild-type reference strain, which increased significantly to >1 in *lem3*Δ/Δ and *rta3*∆/∆ cells. *rta3*Δ/Δ*lem3*Δ/Δ cells exhibited an increase (>1.8) in the ratio, indicating a preference toward the synthesis of C24:0 fatty acids compared to C26:0 (Fig. 2D). Our findings suggest that deletion of *LEM3* causes a stronger perturbation in ergosterol and sphingolipid levels, compared to *RTA3* deletion; however, both genes regulate membrane lipid composition in *C. albicans*.

Next, we presumed that alterations in PC/PE distribution within the lipid bilayer, accompanied by changes in ergosterol and sphingolipid levels in the *lem3*Δ/Δ cells, should affect cell permeability. To assess the consequence of the altered membrane lipid composition on cell permeability, we analyzed all the strains for their abilities to accumulate propidium iodide (PI). A large proportion (>80%) of the *lem3*Δ/Δ and the double mutant accumulated PI after 30 min of exposure with the dye, whereas PI accumulation in *rta3*Δ/Δ cells remained unaltered, compared to the wild type (Fig. S1A). In line with this, both the *lem3*Δ/Δ and the double mutant exhibited a fourfold increase in the number of PI-stained cells, compared to the wild type and the *rta3*Δ/Δ cells, as determined by flow cytometry (Fig. 2E). Colony-forming unit (CFU) analysis revealed no significant loss of viability, indicating that PI staining reflects increased membrane permeability rather than cell death (Fig. S1B). The increase (1.5-fold) in the number of PI-stained cells was also reflected in the *lem3*Δ/Δ and the double mutant, upon amphotericin B treatment, compared to the treated parent strains (Fig. S1C).

Next, we assessed the impact of the altered distribution of PC/PE, ergosterol, and sphingolipids in the *lem3*Δ/Δ and the double mutant on physiological processes dependent on lipid homeostasis, such as endocytosis (30) and activation of the ER stress-induced unfolded protein response (UPR). Using FM4-64, a dye that labels endocytic trafficking to the vacuole, we found no defects in endocytosis in the *lem3*Δ/Δ mutant (Fig. S2). PC and PE are major components of all biological membranes, and disruption in their distribution can induce lipid bilayer stress, leading to ER stress and activation of the UPR (31–33). We therefore asked whether altered membrane lipid composition in *lem3*Δ/Δ cells activates ER stress-induced UPR. The absence of *LEM3* resulted in ER stress, as inferred from the increased susceptibility of the *lem3*Δ/Δ mutant to Tm and dithiothreitol (DTT), compared to the wild type. The double mutant exhibited increased susceptibility to both ER stressors, compared to the *lem3*Δ/Δ mutant, likely due to the combined defects in membrane organization in the absence of *RTA3* and *LEM3*. However, the *lem3*Δ/Δ and the double mutant showed a moderate increase in susceptibility to β-mercaptoethanol (β-ME), compared to the other ER stressors (Fig. 2F). To abate ER stress, *C. albicans* relies on Ire1-Hac1-dependent activation of the UPR, which involves Ire1-mediated splicing of the 19 bp intron from *HAC1* mRNA, resulting in the generation of the Hac1 transcription factor (32). Therefore, UPR activity can be profiled by monitoring the appearance of the spliced *HAC1* (*HAC1^s^*) mRNA. To assess the UPR activity, we monitored the processing of *HAC1* mRNA in wild-type and *lem3*Δ/Δ cells in the absence and presence of Tm (4.75 μM) by performing RT-PCR using primers across the *HAC1* mRNA intron, followed by analyzing the PCR products on an agarose gel. Although compromising Lem3 function created ER stress, the mutant did not trigger constitutive *HAC1* mRNA splicing, as inferred by the absence of the *HAC1^s^* mRNA, in the absence of Tm (Fig. 2G). However, Tm triggered splicing of the *HAC1* mRNA in the mutant, similar to the wild type (Fig. 2G), indicating that the absence of *LEM3* does not affect the activation of the classical UPR pathway. Thus, the sensitivity of the *lem3*Δ/Δ mutant to ER stressors is not due to misregulated UPR activity, as indicated by the lack of basal UPR activity, but to perturbed membrane lipid composition that exacerbates ER stress in the presence of the exogenous ER stressor Tm (Fig. 2F and G). We infer that maintenance of the membrane lipid composition by Lem3 may be required for maintaining ER homeostasis through a mechanism that does not involve the splicing of *HAC1* mRNA. Overall, our findings highlight the dependence of *C. albicans* on the concerted actions of Lem3 and Rta3 for maintaining asymmetric PC/PE distribution across the plasma membrane, a process critical for regulating the levels of other plasma membrane constituents, and for controlling cell permeability.

### Absence of *LEM3* impairs activity of the Cdr1 efflux pump and sensitizes an azole-resistant isolate to fluconazole

**Fig 3:**
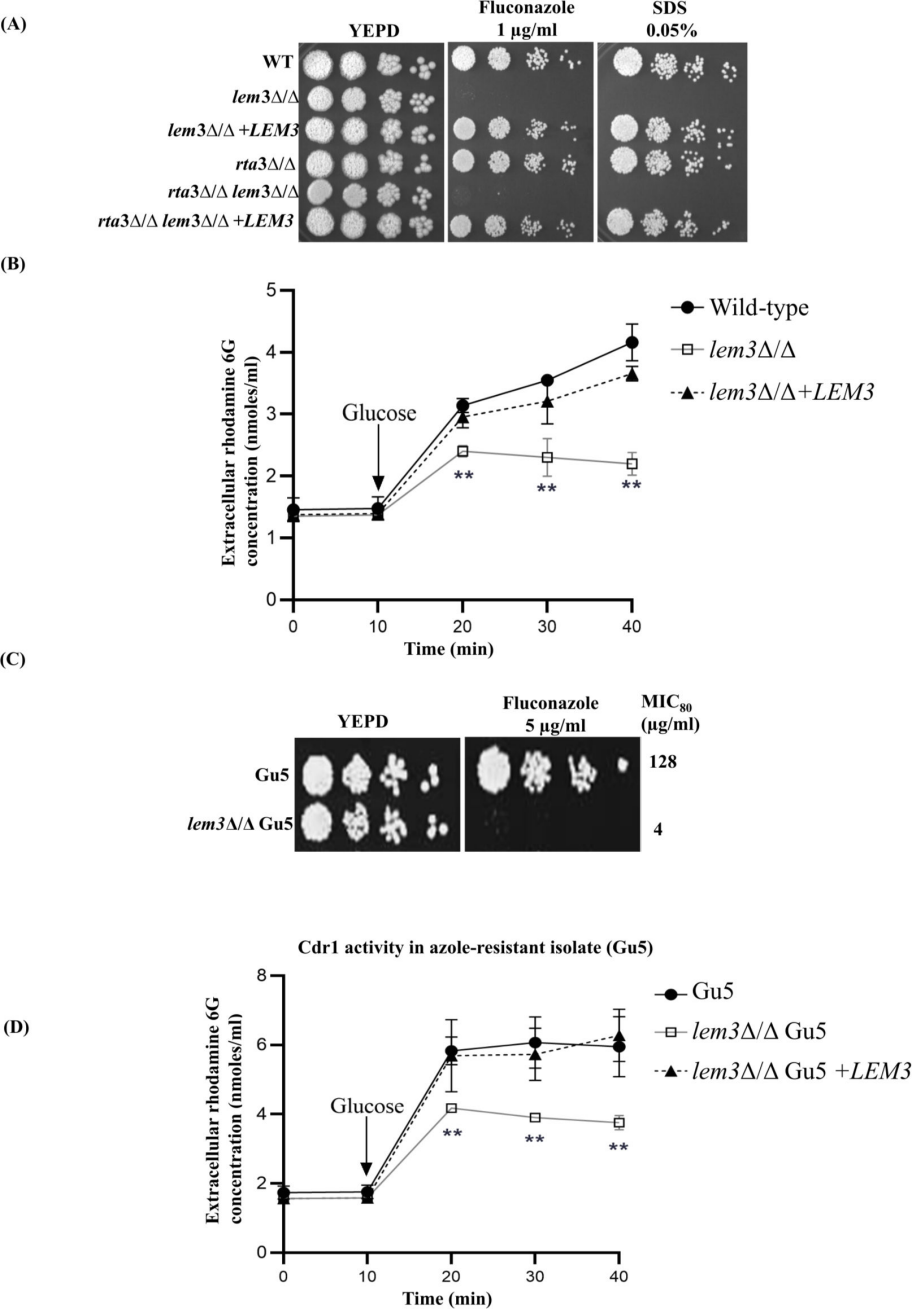
Absence of *LEM3* reverses azole resistance. (**A and C**) Fivefold serial dilutions of strains were spotted on the indicated drug plates and incubated for 48 h at 30°C. (**B and D**) Efflux of rhodamine 6G (R6G) was measured in the indicated strains grown overnight in YEPD, starved for 2 h in PBS, incubated with R6G (10 μM), and then transferred to PBS (pH 7). At 10 min, glucose was added, and efflux of R6G was monitored over 40 min. Data represent means ± SD from three independent assays. **P < 0.01 (Student’s *t* test).

Impaired flippase activity sensitizes pathogenic fungi to multiple stressors (21, 34, 35). Building on this, we examined the growth of the *lem3*Δ/Δ mutant in the presence of various antifungals. The mutant showed increased susceptibility to the azole fluconazole (Fig. 3A). Consistent with its altered membrane permeability, the *lem3*Δ/Δ mutant also failed to grow in the presence of SDS, a membrane-disrupting agent (Fig. 3A).

Given that azole susceptibility in *C. albicans* is directly linked to the activity of the drug efflux pump Cdr1, we examined Cdr1 function in *lem3*Δ/Δ cells using rhodamine 6G (R6G), a substrate of Cdr pumps (36). After the addition of glucose, an increase in the extracellular concentration of R6G from 1.4 to 4 nmol/mL (threefold increase) within 30 min was observed in the wild type and the reconstituted strain. In contrast, the concentration of extracellular R6G increased by only 1.5-fold after the addition of glucose to *lem3*∆/∆ cells, presumably due to reduced activity of Cdr1 (Fig. 3B). Reduction in Cdr1 activity can also be due to its mislocalization in the mutant. To assess this, we used a *CDR1-GFP* construct that was integrated at the native locus in the strains, with its expression driven by the *CDR1* promoter. In both the wild type and the mutant, Cdr1-Gfp was localized to the plasma membrane, as visualized by confocal microscopy (Fig. S3), ruling out mislocalization of the efflux pump as the underlying cause of its reduced activity in the absence of *LEM3*.

Because Lem3 affects Cdr1 function, we investigated whether it modulates drug resistance in an azole-resistant clinical isolate, Gu5 (37). For this, we deleted *LEM3* in Gu5 and tested *lem3*Δ/Δ Gu5 cells for susceptibility to fluconazole. The mutant exhibited increased susceptibility to fluconazole, commensurate with a 32-fold decrease in the MIC_80_ values compared with the parent strain (Fig. 3C). Consistently, the *lem3*Δ/Δ Gu5 cells exhibited an only twofold increase in the extracellular concentration of R6G, compared to its threefold increase in the wild-type and the reconstituted strains, indicating that functional Lem3 is required for maintaining the activity of Cdr efflux pumps in the azole-resistant isolate (Fig. 3D). Overall, these results demonstrate that Lem3-mediated phospholipid flipping affects azole susceptibility through maintenance of Cdr1 efflux pump activity. They also underscore the potential of targeting flippase complex components to modulate drug resistance in *C. albicans*.

### Lem3 impacts the expression of virulence traits

**Fig 4:**
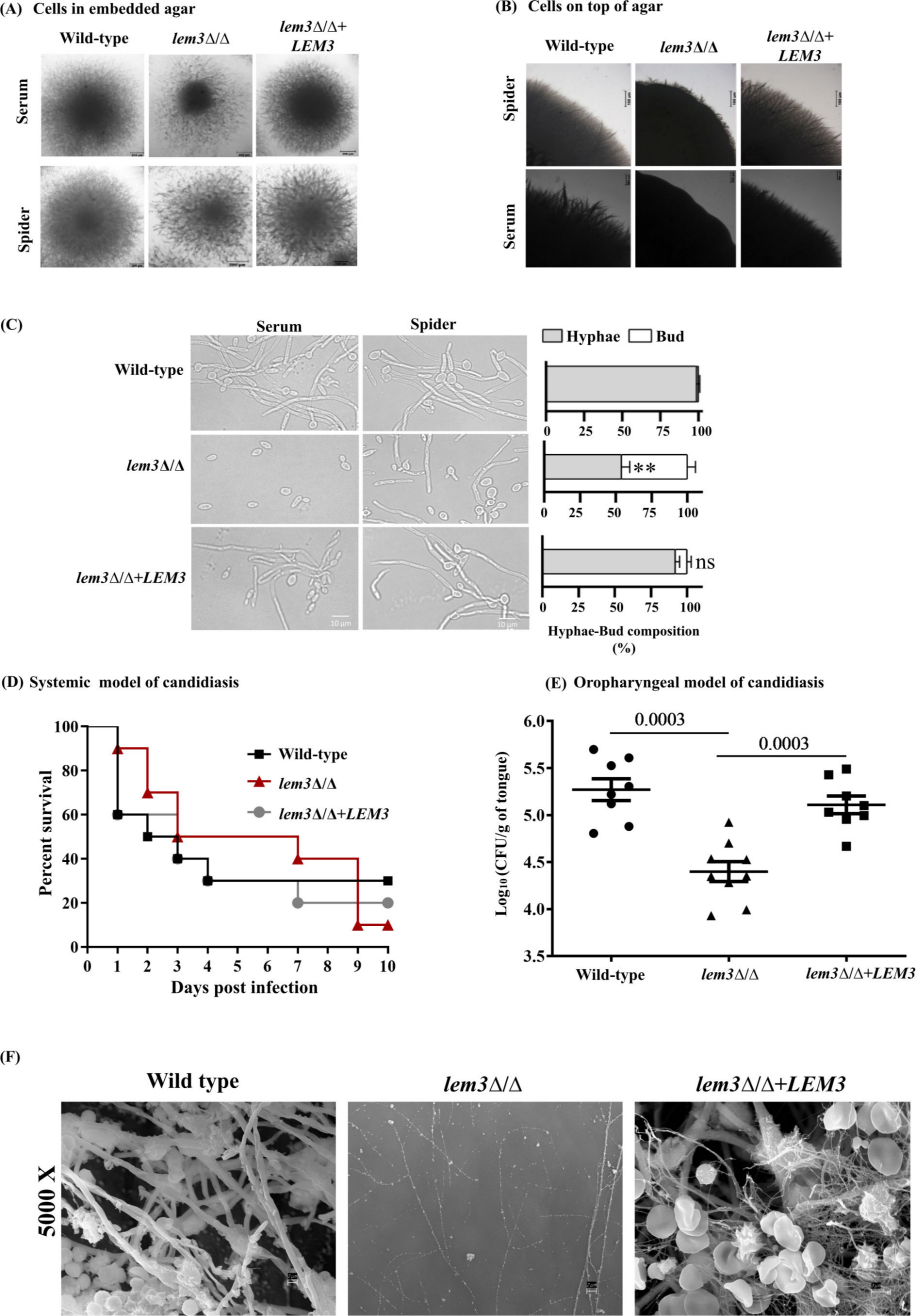
Absence of *LEM3* affects expression of virulence traits. (**A**) The indicated strains were embedded into (**B**) or spotted onto the surface of Spider agar and agar containing 10% FCS, and incubated at 37°C for 7 days. (**C**) Representative images of the indicated strains after 4 h of hyphal induction in filamentation-inducing medium, with the bar graphs on the right indicating the quantified bud-to-hypha ratios for each strain. (**D**) Groups of 10 mice were inoculated via the tail vein with 5 × 10^6^ stationary-phase cells of the indicated cell type and survival monitored daily for 10 days post-inoculation. Kaplan-Meier curves were determined and compared with the log-rank (Mantel-Cox) test. Data represent the mean ± SD from two independent biological replicates. (**E**) Steroid-treated outbred CD1 mice were infected in their oral mucosa with the indicated strains for the OPC model. On day 5 post-infection, mice from the infected strain group were sacrificed. Oral tissues were harvested and processed to determine fungal burden, which was expressed as CFU per gram of tissue weight (*n* = 8 mice per group). Statistical analysis was performed using the Mann-Whitney test, with *P*-values < 0.05 considered statistically significant. (**F**) *In vivo* biofilm formation was examined by inoculating central venous catheters with the indicated strains. The catheters were introduced into rats, incubated for 24 h, and then removed for visualization via scanning electron microscopy (SEM). **P < 0.01; ns; not significant.

Perturbations in plasma membrane lipid composition result in the mislocalization of virulence factors, thus affecting fungal pathogenicity. We tested the ability of the *lem3*Δ/Δ mutant to undergo hyphal morphogenesis under hypha-inducing conditions—Spider as well as serum-containing media. The mutant showed reduced filamentation when embedded within Spider agar (Fig. 4A) or grown on the agar surface (Fig. 4B), whereas serum-induced hypha formation was strongly impaired in both assays (Fig. 4A and B). Next, we performed a time-course experiment in Spider and serum-containing medium to compare filamentation between the strains of interest. At the 2 h time point, both the wild type and the mutant formed hyphae in Spider medium, indicating that Lem3 is not required for hyphal induction (Fig. S4A). However, at 4 h, 54% of the mutant cells remained in the bud form, compared to 2% buds in the wild type (Fig. 4C). A large fraction of the mutant remained in the bud form at later time points, compared to the long-extended hyphae of the reference strains (Fig. S4A), pointing to the requirement of Lem3 in the maintenance and extension of hyphae. The defect in serum-induced hyphal formation was more pronounced, where after 4 h of growth, 1% of cells underwent the bud-to-hypha transition, compared to 95% in the reference strains (Fig. 4C). The mutant continued to exhibit a defect in hyphal formation after 24 h, as evident by the presence of a large proportion of yeast cells (Fig. S4B). It is likely that Lem3 functions downstream or in parallel to canonical hyphal signaling pathways when under strong inducers such as serum. Taken together, these results demonstrate the requirement of Lem3-dependent membrane organization in maintaining hyphal growth.

Thereafter, we tested the mutant for its ability to establish infection in a mouse model of disseminated candidiasis. Mice infected with the *lem3*Δ/Δ mutant survived similarly to the wild type in this model (Fig. 4D). However, in an oropharyngeal candidiasis (OPC) model, the tongues of mice infected with the *lem3*Δ/Δ mutant exhibited approximately one log_10_ reduction in fungal burden compared to the tongues of mice infected with the wild type (Fig. 4E). Next, we tested the *lem3*Δ/Δ mutant for its ability to form biofilms *in vivo*. Implanted catheters in a rat central venous catheter biofilm model were inoculated with strains of interest, and biofilm formation was visualized after 24 h by scanning electron microscopy (SEM) (38). While the wild-type and the reconstituted strains formed normal and mature biofilms, the mutant strain was severely defective for biofilm formation, with few cells attached to the catheter lumen (Fig. 4F).

## DISCUSSION

In this study, we highlight the importance of Lem3, a regulatory subunit of the P4-ATPase lipid flippase complex, in *C. albicans*. Consistent with its predicted transmembrane topology, Lem3 localizes to both the ER and the plasma membrane (Fig. 1B), supporting its direct involvement in lipid transport and in mediating uptake of the alkylphosphocholine drug miltefosine. *C. albicans* Dnf2, the putative catalytic subunit of Lem3, localizes to the plasma membrane at the bud tip when co-expressed with Ca*LEM3* in a *S. cerevisiae dnf1,2*Δ strain, but is retained in the perinuclear and cortical ER in the absence of Ca*LEM3* (16). This indicates that Dnf2-Lem3 likely assembles at the ER, and functional Lem3 is necessary for the transport of the Dnf2-Lem3 complex to the plasma membrane. Exploring the localization of Dnf2 in the absence and presence of Lem3 in a *C. albicans* wild-type strain, and demonstrating a physical interaction between the two proteins, will be necessary to confirm their function as a multisubunit flippase complex.

The phenotypes of the *lem3*Δ/Δ mutant provided evidence that the role of Lem3 in regulating the transbilayer movement of phospholipids across the plasma membrane is well conserved in *C. albicans*. Our finding that the *lem3*Δ/Δ mutant is defective in the internalization (flip) of NBD-labeled PC/PE, in congruence with its decreased susceptibility to miltefosine (Fig. 1C), confirms its role in modulating phospholipid asymmetry. The fact that *CDC50* (*LEM3* paralog) in *S. cerevisiae* (9) and *Candida* species is not involved in the uptake of phospholipids (18, 21) points toward a greater contribution of Lem3 in regulating the inward transbilayer movement of phospholipids in both yeast species. Additional insight into the contribution of each of these regulatory subunits can be gained by creating a combination of mutant strains in *C. albicans*.

The role of the protein kinases Fpk1/Fpk2 in activating the Lem3-Dnf1/Dnf2 complex is well established in *S. cerevisiae* (39). We show that, aside from the kinases, *C. albicans* may also rely on the activity of Rta3, a 7-transmembrane receptor protein for regulating Lem3 activity. An earlier study in *C. albicans* demonstrated a link between Rta3 and uptake of NBD-labeled phospholipids, attributed to Rta3-dependent regulation of an unidentified flippase (24). Sensitivity to miltefosine and enhanced internalization of NBD-PE/PC in the *rta3*Δ/Δ mutant was overridden by the deletion of *LEM3*, suggestive of an epistatic relationship between the two proteins and indicating that Rta3 likely functions upstream of the flippase in *C. albicans* (Fig. 1C). We argue that Rta3 functions as a negative regulator of the transbilayer movement of phospholipids through its effect on Lem3 in *C. albicans*. Whether the regulation is facilitated by a direct interaction between Rta3 and Lem3 warrants further investigation. Owing to its exclusive presence in the fungal kingdom, Rta3 could be exploited as a target for perturbing fungal lipid homeostasis.

A direct relation between flippase activity and ergosterol biosynthesis has been established in *S. cerevisiae*, where deletion of *CDC50* is synthetically lethal in an *erg3*Δ mutant (40). Consistent with this, the *C. albicans lem3*Δ/Δ mutant exhibited altered ergosterol levels (Fig. 2B), linking phospholipid asymmetry and sterol homeostasis; this is also supported by the impact of *C. albicans* Drs2 on the ergosterol distribution (17). Thus, the increased membrane permeability of the *lem3*Δ/Δ mutant, as indicated by an increase in PI uptake, is a summation of the alterations in the levels of phospholipids, ergosterol, and sphingolipids (Fig. 2A through D). Our study also positions Lem3 as a component of the UPR network that contributes to ER stress resistance in *C. albicans* via a Hac1-independent mechanism by maintaining lipid homeostasis that supports ER function. In mammals, phospholipid alterations at the plasma membrane cause premature degradation of the ER-resident Ca^2+^-ATPase ion pump, resulting in depletion of Ca^2+^ stores within the ER (31, 41). As a consequence, chaperones and enzymes dependent on Ca^2+^ for protein folding are rendered inactive, resulting in ER stress and activating the UPR. We posit that an analogous mechanism could be causing ER stress in the *lem3*Δ/Δ mutant, resulting in its increased susceptibility to ER stressors (Fig. 2F). Additionally, the conclusion that targeting Lem3 can reverse drug resistance is based on the finding that the *lem3*Δ/Δ Gu5 cells exhibit abrogated Cdr1 efflux pump activity underlying the 32-fold reduction in MIC_80_ of the azole-resistant Gu5 to fluconazole (Fig. 3C and D). Notably, the absence of the catalytic subunit Dnf2, the putative partner of Lem3, does not alter susceptibility to antifungals, which is in contrast to the *drs2*Δ/Δ and *cdc50*Δ/Δ mutants that exhibited increased susceptibility to azole and echinocandin antifungals (16, 17). The data reveal distinct phenotypic differences between the *lem3*Δ/Δ and *dnf2*Δ/Δ strains, suggesting that *C. albicans* Lem3 associates with multiple catalytic partners such that loss of a single *LEM3* allele perturbs the stoichiometry of these multisubunit complexes, leading to haploinsufficiency (Fig. 1C) (42). Whether Lem3 indeed engages with multiple catalytic partners in *C. albicans* requires further study.

Our findings also forge a link between flippase activity and expression of virulence traits (Fig. 4). The coordinated action of the various flippase complexes ensures maintenance of a lipid gradient at the plasma membrane, pivotal for polarized growth. As an example, PE exposure to the exoplasmic leaflet of the plasma membrane is important for recruitment of Cdc24, the guanine nucleotide exchange factor (GEF) for Cdc42, and Cdc42-mediated activation of cellular processes essential for polarized growth in *S. cerevisiae* (43). It is unlikely that the defective morphogenesis in the *lem3*Δ/Δ mutant directly results from the failed internalization of PE or PC. We presume that, aside from alterations in the membrane lipid composition, as demonstrated in this study, mislocalization of Dnf2 in the absence of Lem3 (16) and misregulation of hyphal growth-specific factors could be contributing to the defect in morphogenesis of the *lem3*Δ/Δ mutant. Strikingly, the non-essentiality of Lem3 during systemic infection suggests that membrane integrity in the mutant remains sufficient to support *C. albicans* survival in blood and deep tissues (Fig. 4D). In contrast, the mutant was specifically defective in causing an infection in the OPC mouse model (Fig. 4E). OPC requires interactions with the oral epithelium, involving efficient adhesion and hypha-mediated invasion to promote colonization of *C. albicans* in the oral cavity (44). Since the mutant did not exhibit an endocytosis defect, it is plausible that functional Lem3 promotes adhesin function through its role in maintaining membrane lipid composition. This also highlights a context-specific requirement for membrane homeostasis during mucosal invasion and points to differences in the fungal requirements for mucosal versus systemic diseases. Compatible with this, impaired internalization of PC/PE together with defective adhesin function may underlie the adherence defect observed *in vivo*, ultimately compromising biofilm formation (Fig. 4F). While the other *C. albicans* flippase mutants (*DRS2*, and *DNF1-3*, *CDC50*) are defective in establishing infection in the mouse model of systemic infection (16–18), their abilities to cause infection in the OPC model or to establish biofilm have not been tested. Altogether, our data support the view that flippases serve as important determinants of plasma membrane asymmetry, drug resistance, and pathogenesis in fungi. Recently, butyrolactol, a natural product, was shown to target Apt1-Cdc50 in *C. neoformans* and sensitize both *C. neoformans* and *Candida* species to echinocandins (45). In this context, our study reinforces the potential of flippases as drug targets and highlights the value of screening for inhibitors as a strategy to develop membrane-directed antifungals.

## MATERIALS AND METHODS

### Strains, chemicals, and growth conditions

Strains, plasmids, and oligonucleotides used in this study are listed in Tables S1 to S3. The strains were grown at 30°C in YEPD (1% yeast extract, 2% peptone, 2% glucose, and 2.5% agar) liquid medium and agar plates, and maintained as frozen stocks. SDC medium (0.67% of yeast nitrogen base [Difco], 2% glucose, and 0.02% complete amino acid supplement) and SC medium (SDC lacking glucose but containing 2% sorbitol) were used for phospholipid internalization assays (27). The transformants were selected on YEPD plates containing 200 μg/mL nourseothricin (Werner Bioreagents). Fluconazole, miltefosine, amphotericin B, myriocin, sodium azide, filipin from Sigma, ER-tracker from Invitrogen, and NBD-PE and NBD-PC from Avanti Polar Lipids (Alabaster, AL) were purchased and added to the media/buffer at concentrations described.

### Strain construction

#### Deletion strain generation

The first and second allele of the *LEM3* gene was disrupted using the *SAT1* flipper in the plasmid pSFS2. For the *LEM3* disruption construct, a 300 bp upstream region of *LEM3* (5′*LEM3^ORF^*) was amplified from SC5314 genomic DNA with primers LEM3P1 and LEM3P2 (Table S3), with introduced KpnI and XhoI restriction sites. A 350 bp *LEM3* downstream region (3′*LEM3*^ORF^) was amplified with primers LEM3P3 and LEM3P4, with introduced SacII and SacI sites. All amplicons (5′*LEM3*^ORF^ and 3′*LEM3*^ORF^) were docked in the pGEMTeasy vector from which they were excised and cloned on both sides of the *SAT1* flipper cassette using the mentioned enzymes. This procedure created the *LEM3* knockout construct plasmid pPA1 (Table S2), which was digested with KpnI and SacI to release the 4.8 kb disruption construct. The wild-type SC5314 strain was electroporated with the disruption cassette, and deletion mutants were selected on plates containing 200 μg/mL nourseothricin (PA10). To obtain nourseothricin-sensitive derivatives of transformants, strains were grown in YPM (1% yeast extract, 2% peptone, and 2% maltose) and plated on 25 μg/mL nourseothricin. A nourseothricin-sensitive heterozygous mutant (PA11) was then used for the second round of transformation, generating the homozygous null mutant strain PA12. The *SAT1* flipper cassette was flipped out from PA12, resulting in PA13. The *LEM3* reconstituted construct was made by amplifying the *LEM3* gene, including upstream and downstream sequences, with primers LEM3.01 and LEM3.04 (Table S3). The *LEM3* downstream region was amplified with primers LEM3.03 and LEM3.02 (Table S3). The PCR products were digested with SacI/SacII and XhoI/ApaI and cloned on both sides of the *SAT1* flipper cassette of pSFS5 (46), to generate pPA2 (Table S2). For the reconstituted strain, the homozygous mutant PA13 was transformed with the insert from pPA2 to yield PA14 (Table S1). Proper integration at each step was confirmed by Southern hybridization.

#### GFP tagging of *LEM3*

The C-terminal GFP-tagging plasmid pADH76, containing GFP preceding the *SAT1* flipper cassette, was used for tagging as previously described (26). The PCR amplicon obtained with primers LEM3myc/GFP-F and LEM3myc/GFP-R comprises GFP fused to the last 65 bp of the *LEM3* ORF without its stop codon, the *SAT1* flipper cassette, and 65 bp of the *LEM3* downstream region. This PCR product was integrated into SC5314 (wild type) to obtain the strain PA31*.* Correct integration was verified by colony PCR using detection primers DET LEM3*-*F, AHO300, DET LEM3*-*R, and AHO301. The primer pairs DET LEM3*-*F and AHO300, and DET LEM3*-*R and AHO302 were used in colony PCR to confirm the flipping out of the *SAT1* flipper cassette. To obtain PA32, the *SAT1* flipper cassette was excised from PA31. The amplicon generated using primers LEM3*-*GFP and AHO283 (Table S3), consisting of the GFP tag and the region of homology to the 3′ end of *LEM3* ORF, was confirmed by sequencing prior to transformation.

#### Overexpression strain construction

The *TDH3-LEM3-GFP* overexpressing *C. albicans* strain (Table S1) was constructed using plasmid pCJN542 (25). Primers LEM3-F-OE-Ag- NAT-Ag-TEF1p and LEM3-R-OE-Ag- NAT-Ag-TDH3p (Table S3) were used to amplify the *Ashbya gossypii TEF1* promoter, the *C. albicans NAT1* ORF, the *A. gossypii TEF1* terminator, and the *C. albicans TDH3* promoter with 100 bp of homology to the *LEM3* promoter region. The transformation of *C. albicans* strains was done as described earlier, and nourseothricin-positive transformants were screened using detection primers LEM3-OE-F-detect and Nat-OE-R-det2-CJN (Table S3), generating PA33.

### Drug susceptibility assays

All strains of interest were grown on YEPD plates overnight. The cells were resuspended in 0.9% saline to an OD_600_ of 0.1. Five microliters of four serial dilutions (5 × 10^3^ to 5 × 10^5^ cells) of each strain was spotted onto YEPD plates in the presence and absence of various stressors. Plates were incubated at 30°C, and growth differences were recorded after 48 h. The broth microdilution method was used as described in CLSI (Clinical and Laboratory Standards Institute) guidelines for assessing minimum inhibitory concentration (MIC_80_). Round-bottomed 96-well microtiter plates were prepared for MIC estimation by adding equal volumes of RPMI-1640 (buffered with 0.165 mol/l MOPS) and serially diluted concentrations of drugs. Then, 100 µL of diluted cell suspensions (10^4^ cells/mL) were added to the wells. The prepared plate with cell dilutions was incubated at 37°C for 48 h. Growth was evaluated by measuring the OD_600_ in a microplate reader and the lowest drug concentration that resulted in 80% inhibition of growth compared with the growth of the drug-free controls was defined as MIC_80_.

### Fluorescence microscopy

The overnight culture was subcultured into fresh YEPD and incubated until the mid-logarithmic phase (OD_600_ ≈ 1.0). For ER-Tracker staining, culture was harvested by centrifugation at 5,000 rpm for 5 min. The resulting cell pellet was resuspended in 1 mL of fresh 1× phosphate-buffered saline (PBS) containing 5 µM ER-Tracker dye (Invitrogen). Cells were incubated in the dark at 37°C for 60 min with agitation at 220 rpm. Following incubation, the stained cells were washed three times with 1× PBS. Thereafter, ER-stained cells were fixed in 4% formaldehyde prepared in PBS and incubated at room temperature for 2 min. For filipin staining, the cells were resuspended in 1× PBS and incubated with 20 µg/mL of filipin in the dark at room temperature for 20 min. Post-staining, the cells were washed three times with PBS to remove unbound dye. Digital images were captured by a confocal microscope using a 100× oil objective lens.

### Internalization of phospholipids into yeast cells

Strains grown to early-log phase (OD_600_ 1.0) in SDC media at 30°C were labeled with DMSO solubilized 5 µM NBD-PC and 20 nM MitoTracker red for 45 min as described previously (27). SDC medium was used to wash the cells three times, followed by incubating the cell suspension at 30°C for an additional 30 min. Final washing (three times) of the cells was done with ice-cold SC-azide. The cells were maintained on ice until further analysis by microscopy or flow cytometry.

### CFU and membrane permeability assays

Overnight cultures of indicated strains were diluted to an OD_600_ of 0.1 in fresh YEPD, followed by withdrawing aliquots at the indicated time points. Tenfold serial dilutions (10^−1^ to 10^−5^) were prepared in PBS, followed by spreading 100 µL of the 10^−5^ dilution on YEPD agar plates. After 48 h of incubation at 30°C, colonies were counted manually. CFU values were calculated as CFU/mL and expressed as log_10_ CFU/mL (mean ± S.D., *n* = 3).

For PI staining, cells grown to the mid-log phase in YEPD were harvested by centrifugation at 5,000 rpm for 5 min and washed twice with sterile 1× PBS. The cell pellet was resuspended in 1 mL of PBS containing 6 µg/mL propidium iodide (PI) and incubated in the dark at room temperature for 20 min. After incubation, the cells were washed three times with PBS to remove unbound dye. Samples were then immediately analyzed using confocal microscopy or flow cytometry (30).

### Flow cytometry assays

For flow cytometry assays, cells were grown to early-log phase (OD_600_ 1.0) in YEPD at 30°C and labeled with the desired fluorophore. A total of 10,000 events were counted, followed by analysis using a BD FACSAria Fusion (Becton Dickinson Immunocytometry Systems, San Jose, CA) equipped with a blue laser emitting at 488 nm (24). Fluorescence was measured on the FL1 fluorescence channel equipped with a 530 nm band-pass filter for NBD-PC and NBD-PE, and on the FL2 channel equipped with a 620/10 nm band-pass filter for PI.

### Efflux of rhodamine 6G

Approximately 10^7^ yeast cells from overnight-grown cultures were inoculated in 50 mL YEPD medium and allowed to grow for 5 h. PBS (pH 7.0) was used to wash the cell pellets twice. 10 μM of Rhodamine 6G (R6G) was added to a 2% cell suspension prepared in 1× PBS. After incubating the cell suspension under glucose starvation conditions for 3 h at 30°C with shaking (200 rpm), the cells were washed, and a 2% cell suspension was prepared in PBS. After the addition of 2% glucose, energy-dependent efflux of R6G was measured. 1 mL of cells was removed at 10-min intervals, centrifuged, and the absorption of supernatants was measured at 527 nm. Glucose-free controls were included in all experiments (36). The amount of effluxed R6G was calculated using a standard concentration curve of R6G.

### Lipid analysis

Ergosterol estimation was performed as described previously (47) with slight modifications. A single colony of *C. albicans* from an overnight YEPD plate was inoculated into YEPD broth. The cultures were incubated at 30°C with shaking at 220 rpm until an optical density (OD_600_) between 2 and 3 was obtained. Subsequently, approximately 5 × 10^8^ cells were harvested, washed twice with sterile-distilled water, and disrupted using glass beads. Cholesterol (Sigma) as an internal standard (25 µg) was added before disruption. An aliquot of 25 µL from the lysate was used for protein estimation to normalize the data. Thereafter, 9 mL of a 2:1 mixture of chloroform:methanol was added to the remaining lysate, and the suspension was incubated for 2 h at 37°C. After discarding the upper layer, 2 mL of 0.9% saline was added and vortexed briefly. Samples were then centrifuged at 7,000 × *g* for 5 min, and the lower layer was transferred to a fresh tube and dried by flushing nitrogen into the tube. Dried extracts were dissolved in 100 μL of derivatizing agent (BSTFA-TMCS [TCI]), followed by incubation at 85°C for 60 min. The net lipid weight was determined after cooling and drying under nitrogen. Gas chromatography-mass spectrometry (GC-MS) analysis was performed on derivatized sterols that were resuspended in chloroform. For the preparation of a standard curve, ergosterol standards of known concentrations, also derivatized with BSTFA-TMCS, were used. The derivatized ergosterol standards and the sample sterols were run through a Restek RTX-5MS Crossbond 5% diphenyl–95% dimethyl polysilane column in a GC-MS (model QP2010 Plus; Shimadzu, Japan). The GC-MS-QP2010 Series software was used for analyzing the retention times and integrated peak areas. The calibration derived from the standard curve of ergosterol was used to determine the ergosterol level, presented as µg of ergosterol per mg of protein weight.

For sphingolipid analysis, overnight-grown cultures were sub-cultured to a starting OD_600_ of 0.1 and allowed to grow until an OD_600_ of 2 was attained. Approximately 50 OD cells were harvested by centrifugation and subjected to lipid analysis as per the protocol described in Kumar et al. with slight modifications (29). Cell pellets washed twice with Milli-Q water were homogenized in 1 mL Mandala buffer (dH_2_O:ethanol:pyridine:diethyl ether:14.2 N ammonium hydroxide; 150:150:10:50:0.18, vol/vol/vol/vol/vol) using glass beads in Fast Prep (MP Biomedicals). C17 ceramide (d18:1/17:0, Avanti Polar Lipids, AL, USA) was added as an internal standard before lysis. Twenty-five microliters of the lysate was used for protein estimation using bicinchoninic acid (BCA) protein assay kit (G-Biosciences, MO, USA), whereas the remaining lysate after incubation at 60°C for 30 min was centrifuged. The supernatant was transferred to another tube and dried by flushing with N_2_ gas. Next, the dried lipid pellet was resuspended in CH_3_OH and CHCl_3_ (2:1) by vigorous vortexing, followed by incubation at 37°C for 1 h, and then centrifuged. The supernatant was transferred to another glass tube, and 1 mL CHCl_3_ and 1 mL dH_2_O were added. After vortexing and centrifugation, the lower layer was separated and transferred to another tube, and then dried with N_2_ flushing. Next, the dried pellet was subjected to alkaline hydrolysis using 0.5 mL of 0.6 M methanolic KOH and 0.5 mL of CHCl_3_, followed by vortexing and incubation at room temperature for 1 h. To this, 0.125 mL dH_2_O and 0.325 mL HCl (1 N) were added and vortexed. The lower layer was dried in a stream of N_2_ gas. For liquid chromatography mass spectrometry, dried samples were dissolved in 0.3 mL organic buffer (1 mM ammonium formate and 0.2% formic acid). From this, a 20 µL sample was diluted 10-fold in the organic buffer. Samples were analyzed by electrospray ionization-based liquid chromatography coupled to a mass spectrometer (QTRAP 4500 AB Sciex, USA) using a C8-column (Waters, USA). The flow program, mass spectrometric parameters, and data analysis were done by methods as described by Ali et al. (47).

### Morphogenesis assays

For the embedding method for analysis of hyphal growth, a single colony from a fresh culture was transferred to YEPD liquid medium and incubated at 30°C in a shaking incubator. Cell density was adjusted to 5 × 10^5^ cells/mL in YEPD broth before incubating for 4 h to achieve log-phase cells. Thereafter, the volume of culture equivalent to 200 cells was added to the spider or 10% fetal calf serum (FCS)-containing agar medium cooled to 50°C. The mixture was poured into a sterile petri dish, allowed to solidify for 1 h, and incubated at 37°C for 7 days (44). For assessing hyphal growth of strains on top of agar, cells from an overnight culture grown in YEPD were washed, and approximately 50–100 cells of each strain were plated on both filamentation-inducing media and incubated at 37°C for 7 days. The plates were photographed using a Zeiss microscope equipped with a digital camera to record filamentation patterns in different strains.

For hyphal growth in liquid media, cells from an overnight culture grown in YEPD were used to subculture from a starting OD_600_ of 0.3 in fresh Spider or (FCS)-containing medium and incubated at 37°C with continuous shaking. Aliquots of the cells were taken out at 2-h intervals for 24 h, washed with 1× PBS, and observed under a light microscope for changes in filamentation patterns.

### Virulence assays

Mice were given access to food and water *ad libitum*. Animal euthanasia followed the Guide for the Care and Use of Laboratory Animals, and humane endpoints were used in survival studies. Swiss Webster mice (wild type) were bred and maintained in our specific pathogen-free animal barrier facility. In all experiments, ten 5- to 7-week-old female mice per group were used. Disease manifestation and survival of mice were monitored daily.

For the mouse model of systemic candidiasis, *C. albicans* cultures for systemic infection were grown for 16 h at 30°C with shaking (225 rpm) in YEPD medium. Prior to mouse inoculation, the overnight cultures were washed three times, enumerated on a hemacytometer, and resuspended in pyrogen-free saline (HyClone; catalog # SH30256.FS). The concentration of *C. albicans* cells was adjusted such that the desired dose was 5 × 10^6^ stationary phase cells per mouse delivered in a volume of 100 μL. Groups of 10 mice per condition were injected via tail vein (i.v.) using a tuberculin syringe with a 25-gauge needle. After inoculation, the mice were observed for 10 days for signs of morbidity (hunched posture, torticollis, weight loss, inactivity, lethargy, and ruffled coat) and mortality. Mice that were significantly moribund were euthanized according to institutionally recommended guidelines, and the date of death was recorded as the following day. Kaplan-Meier curves were generated, and groups were compared using the log-rank (Mantel-Cox) test.

Male, 6-week-old CD1 mice were used for the OPC model. OPC was induced in mice as described previously (48). Mice were injected subcutaneously with cortisone acetate (225 mg/kg of body weight) on days 1 and 3 relative to infection. For inoculation, the animals were sedated, and a swab saturated with 2 × 10^7^ *C. albicans* cells was placed sublingually for 75 min. Mice were sacrificed on day 5 after infection. The tongues were harvested, weighed, homogenized for 30 s, and quantitatively cultured to determine fungal burden, which was expressed as CFU per gram of tissue weight (*n* = 8 mice per group). Statistical analysis was performed using the Mann-Whitney test, with *P*-values < 0.05 considered statistically significant.

### Biofilm assays

#### *In vivo C. albicans* venous catheter biofilm model

A jugular vein rat central venous catheter infection model was used for *in vivo* biofilm studies, as previously described (38). After 24 h of *C. albicans* infection, catheters were removed from the rat. The distal 2 cm of catheter material was removed and subjected to SEM for assaying biofilm growth.

#### Statistical analysis

Data were plotted and analyzed using GraphPad Software. Intergroup comparisons were made using the Student’s *t*-test. *P* value of ≤0.05 was considered significant unless otherwise indicated above.

## SUPPLEMENTAL MATERIAL

Supplemental material spectrum.02965-25-s0001.pdf Fig. S1 to S4; Tables S1 to S3.

ASM does not own the copyrights to Supplemental Material that may be linked to, or accessed through, an article. The authors have granted ASM a non-exclusive, world-wide license to publish the Supplemental Material files. Please contact the corresponding author directly for reuse.
