# Supplementary material for: Lem3, a phospholipid flippase subunit, influences Candida albicans pathogenicity through maintenance of plasma membrane asymmetry
Source: Microbiol Spectr. 2026 Mar 31;14(5):e02965-25. doi: 10.1128/spectrum.02965-25 (PMC13141972; doi:10.1128/spectrum.02965-25)
Supplement: Supplemental material — Fig. S1 to S4; Tables S1 to S3. [file spectrum.02965-25-s0001.pdf]

Figure S1

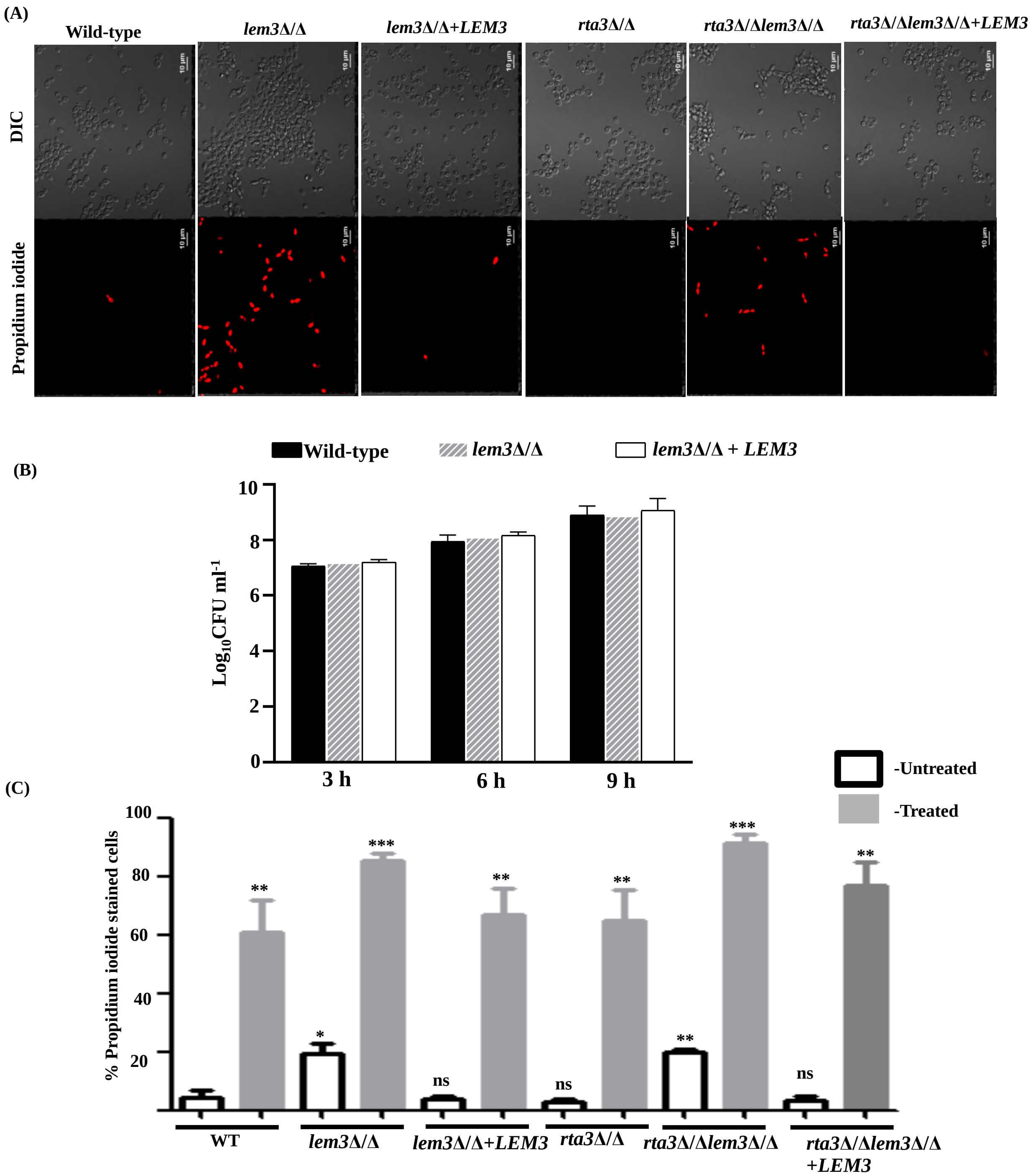

Figure S1. Lem3 regulates plasma membrane integrity. A) Propidium iodide (PI) was added to the indicated *C. albicans* strains and incubated at 30 °C for 20 min. Cells were then washed with PBS and observed by confocal microscopy. **B)** Colony-forming units (CFUs) of the cutures of the indicated strains grown in YEPD, serially diluted, and plated on YEPD agar to determine viable counts (mean ± S.D, n=3). **C)** Cells were grown in YEPD untill OD_{600} 1 and treated for 30 min with 0.25 µg/ml amphotericin B. Both treated and untreated samples were subsequently stained with PI and analyzed by flow cytometry (mean ± S.D, n=3; ns; not significant; ***p<0.01*, ****p<0.001*). Approximately 200 cells were visualized using confocal microscopy.

Figure S2

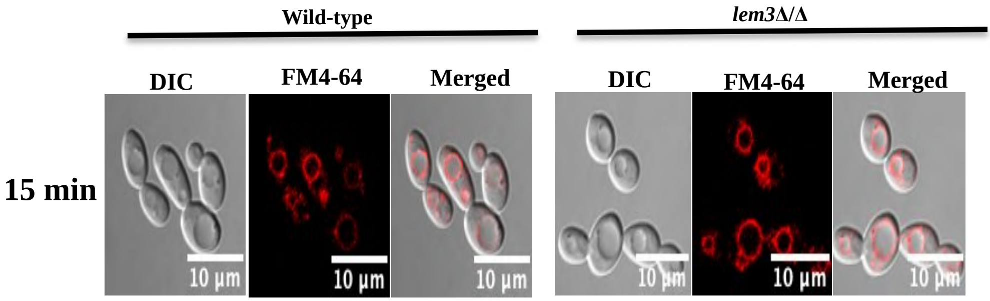

Figure S2. Absence of Lem3 does not impact endocytosis. Strains were grown in in YEPD untill OD_{600} of 1 and incubated with 2 mM FM4-64 for 5, 15 and 30 minutes. At each time point, a cell aliquot visualized using confocal microscopy. Cell visualized at the 15 minute time point are shown.

Figure S3

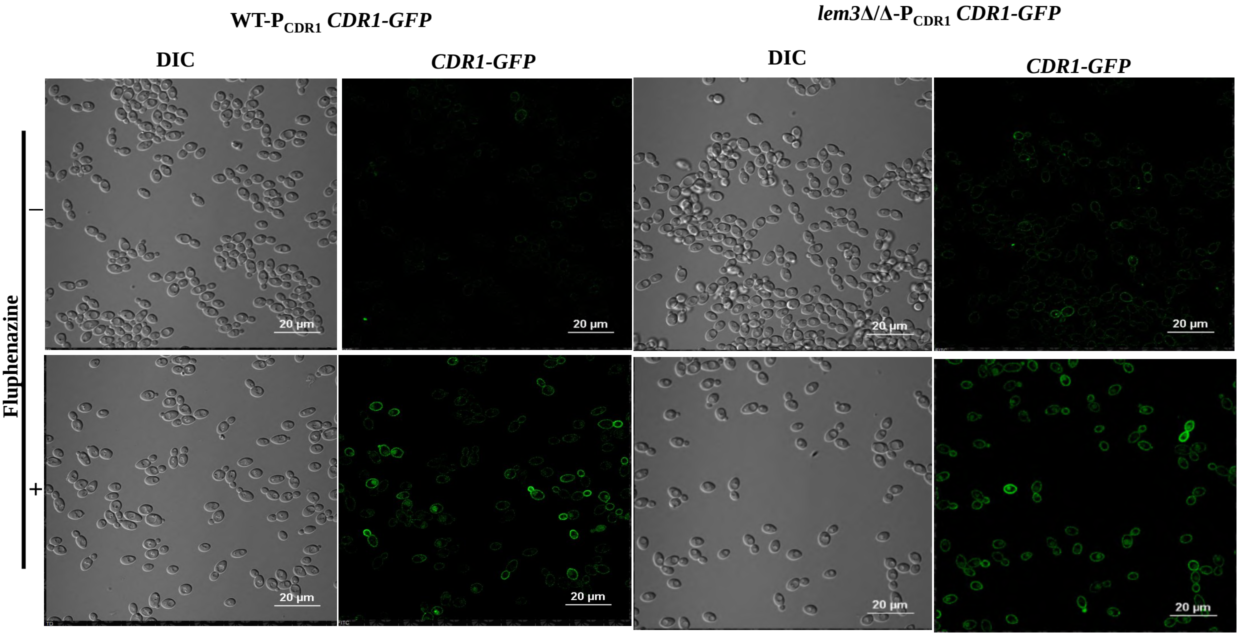

Figure S3. Localization of Cdr1 is unaffected in *lem3*Δ/Δ mutant. Strains were grown in in YEPD until an $OD_{600\ of}$ 1, followed by treating with 20 µg/ml fluphenazine for 30 min before visualization. Approximately 200 cells were visualized using confocal microscopy.

Figure S4

(A) Filamentation in serum-containing medium

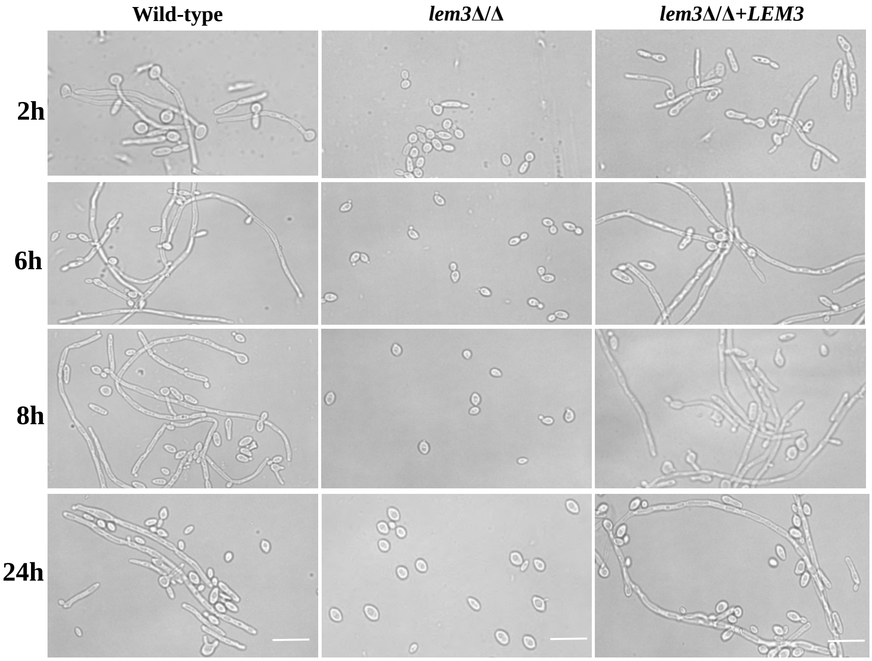

(B) Filamentation in Spider medium

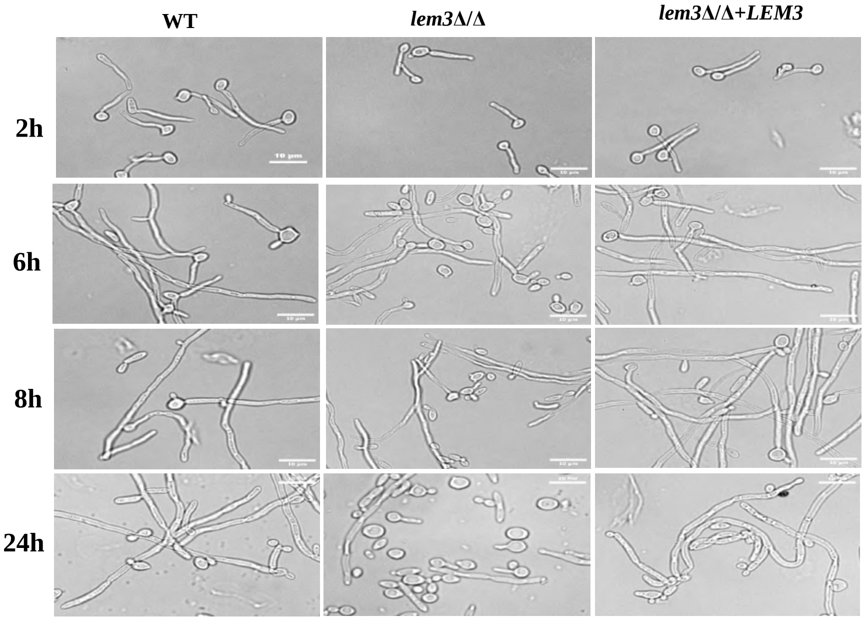

% **Hyphae**

Time (h)	Wild-type	*lem3*Δ/Δ	*lem3*Δ/Δ+ *LEM3*
2	98	93	97
6	93	70	85
8	93	54	77
24	74	47	82

Figure S4. Time-course analysis of hyphal induction. Cells were grown in serum **(A)** (Scale bar is 10 µm), and Spider medium **(B)** for assessing hyphal induction at the indicated time points. The table on the right represents the percentage of hyphal cells quantified microscopically.

SUPPLEMENTARY DATA

SUPPLEMENTARY FIGURE LEGENDS

Figure S1. Lem3 regulates plasma membrane integrity. A) Propidium iodide (PI) was added to the indicated *C. albicans* strains and incubated at 30 °C for 20 min. Cells were then washed with PBS and observed by confocal microscopy. Approximately 200 cells were visualized using confocal microscopy. **B)** Colony-forming units (CFUs) of the cultures of the indicated strains grown in YEPD, serially diluted, and plated on YEPD agar to determine viable counts (mean ± S.D, n=3). **C)** Cells were grown in YEPD untill OD_{600} 1 and treated for 30 min with 0.25 µg/ml amphotericin B. Both treated and untreated samples were subsequently stained with PI and analyzed by flow cytometry (mean ± S.D, n=3; ns; not significant; ***$p<0.01$*, ****$p<0.001$*).

Figure S2. Absence of Lem3 does not impact endocytosis. Strains were grown in YEPD untill OD_{600} of 1 and incubated with 2 mM FM4-64 for 5, 15, and 30 min. At each time point, a cell aliquot was visualized using confocal microscopy. Cell visualized at the 15 min time point are shown.

Figure S3. Localization of Cdr1 is unaffected in *lem3*Δ/Δ mutant. Strains were grown in in YEPD untill OD_{600} 1 and treated for 30 min with 20 µg/ml fluphenazine before visualisation. Approximately 200 cells were visualised using confocal microscopy.

Figure S4. Time-course analysis of hyphal induction. Cells were grown in **(A)** serum (Scale bar is 10 µm), **(B)** and Spider medium for assessing hyphal induction at the indicated time points. The table on the right represents the percentage of hyphal cells quantified microscopically.

SUPPLEMENTARY TABLES

Table S1. Strains used in this study

Strain	Parent	Genotype	Source
SC5314		Wild type	(1)
PA10	SC5314	*LEM3/lem3Δ::SAT1-FLIP*	This study
PA11	PA10	*LEM3/lem3Δ::FRT*	This study
PA12	PA11	*lem3Δ::SAT1-FLIP/lem3Δ::FRT*	This study
PA13	PA12	*lem3Δ::FRT /lem3Δ::FRT*	This study
PA14	PA13	*lem3Δ::FRT/LEM3-SAT1-FLIP*	This study
PA15	PA14	*lem3Δ::FRT/LEM3::FRT*	This study
GU5	GU4	Wild type	(2)
PA18	GU5	*LEM3/lem3Δ::SAT1-FLIP*	This study
PA19	PA18	*LEM3/lem3Δ::FRT*	This study
PA20	PA19	*lem3Δ::SAT1-FLIP/lem3Δ::FRT*	This study
PA21	PA20	*lem3Δ::FRT/lem3Δ::FRT*	This study
PA22	PA21	*lem3Δ::FRT/LEM3-SAT1-FLIP*	This study
AS13	AS12	*rta3Δ::FRT/rta3Δ::FRT* (*rta3Δ/Δ::FRT*)	(3)
PA23	AS13	*rta3Δ/Δ::FRT LEM3/lem3Δ::SAT1-FLIP*	This study
PA24	PA23	*rta3Δ/Δ::FRT LEM3/lem3Δ::FRT*	This study
PA25	PA24	*rta3Δ/Δ::FRT lem3Δ::SAT1-FLIP /lem3Δ::FRT*	This study
PA26	PA25	*rta3Δ/Δ::FRT lem3Δ::FRT /lem3Δ::FRT*	This study
PA31	SC5314	*LEM3:: TDH3P-LEM3-GFP::NAT*	This study

Table S2. Plasmids used in this study

Plasmid	Description	Reference
pSFS2	*SAT1* flipper carrying nourseothricin resistance gene	(4)
pSFS5	pSFS2 carrying the modified *SAT1* flipper cassette	(5)
pPA1	*SAT1* flipper cassette in pSFS2 flanked by 5′ *LEM3* upstream and downstream regions for disruption *LEM3*	This study
pPA2	*LEM3* reconstitution construct	This study
pCJN542	Plasmid for amplifying *TDH3* promoter	(6)
pADH76	C-terminal GFP tagging plasmid	(7)

Table S3. Oligonucleotides used in this study

Oligonucleotide	Description	Sequence*
LEM3P1	Forward primer for amplifying 5′ *LEM3*^{ORF} bearing KpnI site	5'-GGTACCATACAAACTCTGAGCTGA-3'
LEM3P2	Reverse primer for amplifying 5′ *LEM3*^{ORF} bearing XhoI site	5'-CTCGAGCACAAGGATAAATTCTTT-3'
LEM3P3	Forward primer for amplifying 3′ *LEM3*^{ORF} bearing SacII site	5'-CCGCGGAACGTGGTATATACCAAATA-3'
LEM3P4	Reverse primer for amplifying 3′ *LEM3*^{ORF} bearing SacI site	5'-GAGCTCACAATCTTTAATATAGACTTCAA-3'
LEM3.01	To amplify *LEM3* with upstream and downstream sequences bearing SacI site	5'-CTATGAGCTCTTGACTTAATGTTCGAGG-3'
LEM3.04	To amplify *LEM3* with upstream and downstream sequences bearing SacII site	5'-TTTGCCGCGGGAAGACAAAACTGTTACA-3'
LEM3.03	To amplify *LEM3* downstream sequences bearing XhoI site	5'-AAAACTCGAGTTTATTTTGAGTATGAATTCT CTTTG-3'
LEM3.02	To amplify *LEM3* downstream sequences bearing ApaI site	5'-TTTGGGGCCCGAAGACAAAACTGTTACAC-3'
LEM3myc/GFP-F	To amplify 65 bp *LEM3* ORF with GFP tag	5'-AGAAGACAAAGAGAGATGAACAAAGTGCTG CAGCTGCTGAGGGTGTCACCACTGGATTTGAAA AACGGATCCCCGGGTTAATTAACGG-3'
LEM3myc/GFP-R	To amplify 65 bp *LEM3* UTR with *GFP* tag	5'-CCAACATTTATTACAACACAATCTTTAATA TAGACTTCAAAGAGAATTCATACTCAAAATA AACTGGCGGCCGCTCTAGAACTAGTGGATC-3'

DET LEM3-F	To detect *LEM3-GFP* construct integration	5'-ACAAAAATAGATTTAAGAAA-3'
DET LEM3-R	To detect *LEM3-GFP* construct integration	5'-ATTGTACCTTGAAAGAACGG-3'
AHO300	To detect *LEM3-GFP* construct integration	5'-CCGTTAATTAACCCGGGGATC-3'
AHO301	To detect *LEM3-GFP* construct integration	5'-GGAACTTCAGATCCACTAGTTCTAGAGC-3'
AHO302	To detect integration of *LEM3-GFP*	5'-TCACTAGTGAATTCGCGCTCGAG-3'
AHO283	To detect integration of *LEM3-GFP* integration	5'-GGCGGCCGCTCTAGAACTAGTGGATC-3'
LEM3-GFP (150 bp upstream of stop codon)	To detect integration of *LEM3-GFP*	5'-TTTATATTAGGATTAGCCTT-3'
LEM3-F-OE-Ag-NAT-Ag-TEF1p	Forward primer for *LEM3* overexpression (*LEM3*^{OE})	5'-GACAAAAGTTCTTATACTAAGGGGACGATC GGATATATTTTTCCAACCAATAGGACAGATAC CAGGACACATAGCCCCTGTTTCATTATCCCCAT AATCATCAAGCTTGCCTCGTCCCC- 3'
LEM3-R-OE-Ag-NAT-Ag-TDH3p	Reverse primer for *LEM3*^{OE}	5'-ATGATTCATATTCTCCCTCCTGTGCATACTCA TCTAATACACCTTCTCGCTGTTGATCTCCATTAT CAATTTGTTGTGCATCATCTGCTGTTCGTGACA TATTTGAATTCAATTGTGATG-3'
LEM3-OE-F-detect	Detection primer for overexpression	5'-AGCTTAACACGACTGAACAG-3'
HAC1SP-F	*For HAC1* intron splicing determination	5'-AGACGCTTTTAATTACCACACCA-3'
HAC1SP-R	For *HAC1* intron splicing determination	5′-TCAAAGTCCAACTGAAATG-3'
Nat-OE-R-det2-CJN	Detection primer for *LEM3*^{OE}	5'-GAAACAACAACGAAACCAGC-3'
ACT1/RT/F	Primer for qPCR	5'-GAAGCCCAATCCAAAAGAGG-3'

ACT1/RT/R	Primer for qPCR	5'-CTTCTGGAGCAACTCTCAAT-3'
LEM3/RT/F	Primer for qPCR	5'-ATGTCACGAACAGCAG-3'
LEM3/RT/R	Primer for qPCR	5'-CTCGTCATCGTAATCTG-3'

* Restriction sites used in the primers are underlined.

References to Supplementary Data

1. Gillum AM, Tsay EY, Kirsch DR. 1984. Isolation of the *Candida albicans* gene for orotidine-5′-phosphate decarboxylase by complementation of *S. cerevisiae* ura3 and *E. coli* pyrF mutations. Mol Gen Genet 198:179–182.
2. Franz R, Kelly SL, Lamb DC, Kelly DE, Ruhnke M, Morschhäuser J. 1998. Multiple molecular mechanisms contribute to a stepwise development of fluconazole resistance in clinical *Candida albicans* strains. Antimicrob Agents Chemother 42:3065–3072.
3. Srivastava A, Sircaik S, Husain F, Thomas E, Ror S, Rastogi S, Alim D, Bapat P, Andes DR, Nobile CJ. 2017. Distinct roles of the 7-transmembrane receptor protein Rta3 in regulating the asymmetric distribution of phosphatidylcholine across the plasma membrane and biofilm formation in *Candida albicans*. Cell Microbiol 19:e12767.
4. Reuß O, Ashild V, Kolter R, Morschhäuser J. 2004. The *SAT1* flipper, an optimized tool for gene disruption in *Candida albicans*. Gene 341:119–127.
5. Sasse C, Schillig R, Dierolf F, Weyler M, Schneider S, Mogavero S, et al. (2011). The transcription factor Ndt80 does not contribute to Mrr1-, Tac1-, and Upc2-mediated fluconazole resistance in *Candida albicans*. PLoS ONE 6(9): e25623
6. Nobile CJ, Schneider HA, Nett JE, Sheppard DC, Filler SG, Andes DR, Mitchell AP. 2008. Complementary adhesin function in *C. albicans* biofilm formation. Curr Biol 18:1017–1024.
7. Lohse MB, Johnson AD. 2016. Identification and characterization of Wor4, a new transcriptional regulator of white-opaque switching. G3 (Bethesda) 6:721–729.
